# Risk factors for candidemia: a prospective matched case-control study

**DOI:** 10.1186/s13054-020-2766-1

**Published:** 2020-03-18

**Authors:** Julien Poissy, Lauro Damonti, Anne Bignon, Nina Khanna, Matthias Von Kietzell, Katia Boggian, Dionysios Neofytos, Fanny Vuotto, Valérie Coiteux, Florent Artru, Stephan Zimmerli, Jean-Luc Pagani, Thierry Calandra, Boualem Sendid, Daniel Poulain, Christian van Delden, Frédéric Lamoth, Oscar Marchetti, Pierre-Yves Bochud, the FUNGINOS, J. D. Aubert, Ch. Berger, P.-Y. Bochud, K. Boggian, T. Calandra, A. Christe, A. Conen, C. Corti-Fragoso, P. Dutkowski, Ph. Eggimann, C. Garzoni, D. Goldenberger, N. Khanna, F. Lamoth, O. Marchetti, E. Marques Maggio, K. Mühlethaler, D. Neofytos, D. Sanglard, P. W. Schreiber, U. Schanz, C. van Delden, M. Von Kietzell, R. Zbinden, S. Zimmerli, Allfun French Study Groups, F. Artru, A. Bignon, V. Coiteux, D. Deblauw, A. El Kalioubie, K. Faure, N. François, T. Galpérine, B. Guéry, E. Jaillette, E. Kipnis, D. Mathieu, C. Nilès, E. Parmentier-Decrucq, J. Poissy, D. Poulain, S. Préau, L. Rahmania, L. Robriquet, A. Rouzé, B. Sendid, E. Vega, B. Voisin, P. Weyrich

**Affiliations:** 1 Current affiliation : Univ. Lille, Inserm U1285, CHU Lille, Pôle de réanimation, NRS, UMR 8576 - UGSF - Unité de Glycobiologie Structurale et Fonctionnelle, F-59000 Lille, France; 2 Inserm, U995-2 “Fungal Associated Invasive and Inflammatory Diseases”, F-59000 Lille, France; 3 Infectious Diseases Service, Department of Medicine, Lausanne University Hospital and University of Lausanne, rue du Bugnon 46, CH-1011 Lausanne, Switzerland; 4 Department of Infectious Diseases Department, Inselspital, Bern University Hospital, University of Bern, Bern, Switzerland; 5 Surgical Intensive Care Unit, University Hospital of Lille, F-59000 Lille, France; 6 Division of Infectious Diseases and Hospital Epidemiology, University and University Hospital of Basel, Basel, Switzerland; 7 Infectious Diseases Department, Cantonal Hospital of Saint Gallen, St. Gallen, Switzerland; 8 Transplant Infectious Diseases Unit, University Hospitals of Geneva, Geneva, Switzerland; 9 Infectious Diseases Department, University Hospital of Lille, F-59000 Lille, France; 10 Hematological Disorders Department, University Hospital and University of Lille, F-59000 Lille, France; 11 Digestive Intensive Care Department, University Hospital and University of Lille, F-59000 Lille, France; 12 Adult Intensive Care Service, University Hospital and University of Lausanne, Lausanne, Switzerland; 13 Laboratory of Mycology and Parasitology, Hospital and University of Lille, F-59000 Lille, France; 14 Microbiology Institute, Lausanne University Hospital and University of Lausanne, CH-1010 Lausanne, Switzerland; 15 Department of Medicine, Ensemble Hospitalier de la Côte, CH-1110 Morges, Switzerland

**Keywords:** Candidemia, Risk factors, Central venous catheter, Total parenteral nutrition, Scores, Antibiotics

## Abstract

**Background:**

Candidemia is an opportunistic infection associated with high morbidity and mortality in patients hospitalized both inside and outside intensive care units (ICUs). Identification of patients at risk is crucial to ensure prompt antifungal therapy. We sought to assess risk factors for candidemia and death, both outside and inside ICUs.

**Methods:**

This prospective multicenter matched case-control study involved six teaching hospitals in Switzerland and France. Cases were defined by positive blood cultures for *Candida* sp. Controls were matched to cases using the following criteria: age, hospitalization ward, hospitalization duration, and, when applicable, type of surgery. One to three controls were enrolled by case. Risk factors were analyzed by univariate and multivariate conditional regression models, as a basis for a new scoring system to predict candidemia.

**Results:**

One hundred ninety-two candidemic patients and 411 matched controls were included. Forty-four percent of included patients were hospitalized in ICUs, and 56% were hospitalized outside ICUs. Independent risk factors for candidemia in the ICU population included total parenteral nutrition, acute kidney injury, heart disease, prior septic shock, and exposure to aminoglycoside antibiotics. Independent risk factors for candidemia in the non-ICU population included central venous catheter, total parenteral nutrition, and exposure to glycopeptides and nitroimidazoles. The accuracy of the scores based on these risk factors is better in the ICU than in the non-ICU population. Independent risk factors for death in candidemic patients included septic shock, acute kidney injury, and the number of antibiotics to which patients were exposed before candidemia.

**Discussion:**

While this study shows a role for known and novel risk factors for candidemia, it specifically highlights important differences in their distribution according to the hospital setting (ICU versus non-ICU).

**Conclusion:**

This study provides novel risk scores for candidemia accounting for the hospital setting and recent progress in patients’ management strategies and fungal epidemiology.

## Take home message

The epidemiology of candidemia is changing with the constant evolution of medical and surgical care. In this study, we show that the risk of candidemia depends on exposure to different antibiotics and/or medical procedures in ICU and non-ICU patients, highlighting the need for setting-specific risk assessment scores.

## Introduction

*Candida* spp. are the third most common microorganisms responsible for health-care-related bloodstream infections [1]. The incidence of candidemia has increased by 50% over the last decade worldwide and ranges between 2.4/100000 and ~ 15/100000 individuals, depending on the country and clinical setting [2–6]. Despite significant progress in antifungal treatment options, candidemia is still associated with an overall crude mortality rate ranging between 40 and 60% [4, 7–11]. Attributable mortality ranges from 5% to 49% [12–14], depending on the control group considered and the underlying comorbidities, the impact of nosocomial infections being known to be greater in less sick population, and so probably less important in ICU patients [15]. Prompt initiation of appropriate antifungal therapy is crucial to improve the chances of survival [16]. However, blood cultures for yeasts lack sensitivity and need prolonged incubation (> 24 h). As a consequence, antifungal drugs are often prescribed either prophylactically, pre-emptively, or empirically in high-risk patients [17]. The resulting overuse of antifungal drugs may lead to the emergence of *Candida* species that are resistant to azoles and/or echinocandins [5, 18–20].

Few studies used a matched case-control design to assess risk factors for candidemia [21–25]. Unmatched studies identified factors such as a central venous catheter (CVC), prior surgery, broad-spectrum antibiotic therapy, or total parenteral nutrition (TPN) which are present in a large number of hospitalized patients [22, 23, 26–28]. Furthermore, most studies were performed either inside or outside intensive care units (ICUs) and a few of them allowed for differential analyses according to both settings [24]. This prospective, multicenter, matched case-control study aims to assess the risk factors associated with candidemia in high-risk groups of patients in both the ICU and non-ICU settings.

## Materials and methods

### Study design and patients

This multicenter, international, prospective, matched case-control study was carried out in five university hospitals (Lille, France; Lausanne, Geneva, Bern, and Basel, Switzerland) and a large teaching hospital (St. Gallen, Switzerland) contributing to the Fungal Infection Network of Switzerland (FUNGINOS)—and ALLFUN networks between July 2013 and March 2017. Patients were included if they were > 18 years old with at least one blood culture positive for *Candida* spp. Matched controls (up to three per case) were selected by local investigators for each case. Matching criteria included age (+/− 5 years), hospital ward, duration of hospital stay (time from hospital admission to candidemia in each case was matched to a length of hospitalization at least equal for the corresponding control; most controls remained hospitalized after their inclusion, they were followed-up to ensure that they did not develop candidemia), and the type of surgery in case of surgical procedure. Patients with a history of intravenous drug abuse were excluded from the study as they usually have a clinical risk profile that is different from other candidemic patients.

### Laboratory tests

Two automated blood culture systems were used during the study period: Bactec™ (Becton Dickinson, Sparks, Maryland, USA) and Bact/Alert®3D (bioMérieux, Marcy l’Etoile, France). Yeasts isolated from blood cultures were identified by MALDI-TOF mass spectrometry (Microflex Mass Spectrometer, Bruker Daltonics GmbH, Bremen, Germany) as described previously [29]. Isolates with MALDI-TOF score less than 1.7 were subsequently identified by molecular methods, as reported previously [30].

### Data collection and definitions

Demographic characteristics and underlying medical conditions were recorded systematically for each case and matched controls in a secured electronic case report form (eCRF). Corticosteroid use was defined by the use of > 20 mg prednisone-equivalent daily for > 10 days before positive blood cultures. Clinical conditions and risk factors within 2 weeks prior to candidemia (or a matched time in controls) were also recorded, including the presence of intravenous and urinary devices, TPN, mechanical ventilation (for > 24 h), renal replacement therapy, and use of gastric acid secretion inhibitors. The use of antibacterial and antifungal drugs within 4 weeks before candidemia (or equivalent time in controls) was also recorded. Whenever available, *Candida* colonization index and *Candida* score were recorded by using the method described by Pittet et al. [31] and Léon et al. [22, 27]. We defined ICU population as patients hospitalized in ICU at the time of candidemia and conversely for non-ICU population.

### Statistical analysis

Statistical analyses were performed using the Stata software (v15.1; College Station, TX, USA). Factors associated with candidemia and mortality were analyzed by using univariate and multivariate conditional logistic regression models. A backward stepwise logistic regression was used to select variables entered in the multivariate models, using a cutoff *p* value of 0.10. New scores to predict the risk of candidemia were developed for patients in and outside the ICU. Scores were obtained by rounding the β-coefficients. Receiving operating characteristic (ROC) curves were drawn using rocreg implemented in Stata®, after adjustment for matching covariates [32]. Test efficiencies were calculated using the dtroc softwares (Stata®). The best cutoff point was established according to standard methods (Youdden’s approach to determine the cutoff with the best compromise between sensitivity and specificity; the method of Zweig and Campbell, maximizing efficiency) [33, 34] by using cutpt (Stata®).

## Results

### Study population

The study included 192 patients with candidemia and 411 controls matched for age, hospital duration stay, ward, and type of surgery in case of surgery. Patients were almost equally distributed between surgical (56%) and medical wards (44%) and between non-ICU (53%) and ICU (47%). Median age was 63 years [52–74] and approximately two-thirds of patients were male. Candidemia occurred within a median duration of 16 days (interquartile range 5–27) after hospital admission. *Candida albicans* was the most commonly reported species (61%), followed by *Candida glabrata* (16%), *Candida parapsilosis* (9%), *Candida tropicalis* (3%), *Candida krusei* (3%), and other/mixed species (8%).

### Risk factors for candidemia

Univariate and multivariate analyses of risk factors for candidemia according to hospital setting are shown in Table 1 and in Table 2, respectively. Independent risk factors for candidemia in the whole population included central venous catheter (OR = 6.74, 95% confidence interval (CI) 2.96–15.4, *p* < 0.001), TPN (OR = 3.92, 95%CI 2.28–6.73, *p* < 0.001), previous septic shock (OR = 2.29, 95%CI 1.33–3.96, *p* = 0.003), exposure to nitroimidazoles (OR = 2.16, 95%CI 1.11–4.21), and renal replacement therapy (OR = 2.16, 95%CI 1.11–4.21, *p* = 0.02).

**Table 1:** Demographic and clinical characteristics of patients with candidemia and matched controls inside and outside intensive care units

Characteristics	Whole population			Intensive care			Non-intensive care		
	Controls (*n* = 411)	Cases (*n* = 192)	*p*	Controls (*n* = 172)	Cases (*n* = 83)	*p*	Control (*n* = 239)	Cases (*n* = 109)	*p*
Underlying medical conditions									
Heart disease	321 (78%)	159 (83%)	**0.05**	143 (83%)	76 (92%)	**0.02**	178 (74%)	83 (76%)	0.15
Acute kidney injury	77 (19%)	55 (29%)	**0.002**	45 (25%)	43 (52%)	**< 0.001**	32 (13%)	12 (11%)	0.40
Respiratory disease	84 (20%)	31 (16%)	0.18	42 (24%)	20 (24%)	0.90	42 (18%)	11 (10%)	0.08
Diabetes	81 (20%)	49 (26%)	**0.03**	36 (21%)	23 (28%)	0.12	45 (19%)	26 (24%)	0.12
Solid cancer	81 (20%)	41 (21%)	0.60	20 (12%)	13 (16%)	0.40	61 (26%)	28 (26%)	0.12
Central nervous system disease	50 (12%)	30 (16%)	0.09	22 (13%)	14 (17%)	0.17	28 (12%)	16 (15%)	0.30
Liver disease	36 (9%)	20 (10%)	0.30	14 (8%)	12 (14%)	0.06	22 (9%)	8 (7%)	0.60
Solid organ transplant	24 (6%)	9 (5%)	0.70	10 (6%)	6 (7%)	0.70	14 (6%)	3 (3%)	0.30
Onco-hematological disease	21 (5%)	10 (5%)	0.40	3 (2%)	1 (1%)	1.00	18 (8%)	9 (8%)	0.60
Neutropenia	15 (4%)	11 (6%)	0.30	3 (2%)	0 (0%)	–	12 (5%)	11 (10%)	0.08
Immunosuppressive drugs^1^	57 (14%)	26 (14%)	1.00	18 (10%)	13 (16%)	0.40	39 (16%)	13 (12%)	0.40
Corticosteroids^1^	44 (11%)	22 (11%)	0.90	14 (8%)	13 (16%)	0.20	30 (13%)	9 (8%)	0.30
Other^1^	34 (8%)	16 (8%)	0.90	11 (6%)	8 (10%)	0.30	23 (10%)	8 (7%)	0.70
Other immunosuppression^2^	5 (1%)	10 (5%)	0.02	3 (2%)	5 (6%)	0.10	2 (1%)	5 (5%)	0.11
SAPS^3^	NA	NA	NA	50 [34–62]	58 [40–70]	**0.006**	NA	NA	**NA**
Hospital management and clinical risk factors^4^									
Antacids	309 (75%)	156 (81%)	0.19	141 (82%)	68 (82%)	0.70	168 (70%)	88 (81%)	0.06
Central venous catheter	269 (65%)	170 (89%)	**< 0.001**	149 (87%)	80 (96%)	**0.01**	120 (50%)	90 (83%)	**< 0.001**
Urinary catheter	259 (63%)	137 (72%)	**0.03**	150 (88%)	77 (93%)	0.20	109 (46%)	60 (56%)	0.07
Invasive mechanical ventilation^5^	146 (36%)	75 (39%)	0.20	113 (66%)	69 (83%)	**0.018**	33 (14%)	6 (6%)	**0.04**
Renal replacement therapy^6^	47 (11%)	44 (23%)	**< 0.001**	29 (17%)	36 (43%)	**< 0.001**	18 (8%)	8 (7%)	0.60
Total parenteral nutrition	55 (13%)	77 (40%)	**< 0.001**	27 (16%)	38 (46%)	**< 0.001**	28 (12%)	39 (36%)	**< 0.001**
Antifungal prophylaxis^7^	22 (5%)	20 (10%)	**0.02**	11 (6%)	8 (10%)	0.40	11 (5%)	12 (11%)	**0.02**
Previous septic shock	71 (17%)	68 (35%)	**< 0.001**	40 (23%)	45 (54%)	**< 0.001**	31 (13%)	23 (21%)	**0.02**
Intraabdominal bacterial infection	52 (13%)	33 (17%)	0.11	13 (8%)	15 (18%)	**0.02**	39 (16%)	18 (17%)	0.90
Laboratory data (median, interquartile range, IQR)									
Leucocyte count (10^3^/mm^3^)	14 (9–21)	14 (8–22)	0.70	17 (11–24)	19 (13–27)	0.70	12 (8–18)	10 (7–17)	0.50
C-reactive protein (mg/L)	122 (41–240)	161 (88–266)	**0.003**	149 (75–252)	183 (94–267)	0.14	89 (19–214)	148(72–263)	**0.006**
Bêta-D-glucan (pg/mL)	39 (0–115)	111 (30–348)	**0.03**	39 (0–112)	96 (30–298)	0.06	40 (0–288)	121 (36–450)	0.30
Median colonization index^8^	NA	NA	NA	1 (0–1)	1 (1–1)	0.06	NA	NA	NA
Median corrected colonization index^8^	NA	NA	NA	0 (0–0)	0 (0–1)	0.14	NA	NA	NA
Median candida score^8^	NA	NA	NA	2 (1–2)	3 (2–4)	**0.02**	NA	NA	NA
Antibacterial therapy^7^									
Antibiotics (any)	310 (75%)	174 (91%)	**< 0.001**	154 (90%)	79 (95%)	0.11	156 (65%)	95 (87%)	**< 0.001**
Number of antibiotics(median, IQR)	2 [1–3]	2 [1–4]	**< 0.001**	2 [1–4]	3 [2–4]	**< 0.001**	1 [0–2]	2 [1–3]	**0.03**
Amoxicilline/clavulanate	66 (16%)	27 (14%)	0.90	39 (23%)	18 (22%)	0.80	27 (11%)	9 (8%)	0.60
Pipéracilline/tazobactam or ticarcilline/clavulanate	155 (38%)	99 (52%)	**0.003**	74 (43%)	53 (64%)	**0.008**	81 (34%)	46 (42%)	0.13
Cephalosporins G1/2	36 (9%)	21 (11%)	0.30	27 (16%)	15 (18%)	0.30	9 (4%)	6 (6%)	0.50
Cephalosporins G3	62 (15%)	24 (13%)	0.60	28 (16%)	11 (13%)	0.70	34 (14%)	13 (12%)	0.70
Cephalosporins G4	34 (8%)	19 (10%)	0.30	19 (11%)	12 (14%)	0.40	15 (6%)	7 (6%)	0.70
Carbapenems	75 (18%)	60 (31%)	**0.001**	43 (25%)	32 (39%)	**0.03**	32 (13%)	28 (26%)	**0.008**
Fluoroquinolones	58 (14%)	35 (18%)	0.12	31 (18%)	22 (27%)	**0.05**	27 (11%)	13 (12%)	0.90
Glycopeptides	56 (14%)	45 (23%)	**0.006**	34 (20%)	22 (27%)	0.40	22 (9%)	23 (21%)	**0.002**
Sulfamides	16 (4%)	9 (5%)	0.80	5 (3%)	7 (8%)	0.14	11 (5%)	2 (2%)	0.20
Nitroimidazoles	33 (8%)	23 (12%)	**0.05**	17 (10%)	11 (13%)	0.30	16 (7%)	12 (11%)	0.06
Aminoglycosides	77 (19%)	46 (24%)	**0.03**	44 (26%)	31 (37%)	**0.01**	33 (14%)	15 (14%)	0.70

*NA* not adapted

^1^Corticosteroids were considered for > 20 mg equivalent prednisone during more than 10 days. Other immunosuppressive drugs include methotrexate, aziathoprine, tacrolmus, and sirolimus

^2^HIV and asplenia. Two HIV patients in cases, exclusively in ICU

^3^Simplified Acute Physiology Score, available only for ICU patients

^4^Within 2 weeks before candidemia (cases) or matched time period (controls)

^5^Invasive mechanical ventilation for ≥ 24 h. Some patient in general ward are included as they were had mechanical ventilation during a previous stay in an ICU

^6^Chronic and/or acute extra renal epuration

^7^Within 4 weeks before candidemia (cases) or matched time period (controls)

^8^Vailable for 38 cases and 30 controls, in ICU

**Table 2:** Independent risk factors associated with candidemia according to hospitalization inside and outside intensive care units

Risk factors	Whole population^1, 2^ (*N* = 567)			Intensive care^1, 2^ (*N* = 250)			Non-Intensive care^1, 2^ (*N* = 322)		
	OR	95% CI	*p*	OR	95% CI	*p*	OR	95% CI	*p*
Central venous catheter^4^	6.74	2.96–15.4	< 0.001				9.77	3.72–25.7	< 0.001
Total parenteral nutrition^4^	3.92	2.28–6.73	< 0.001	6.75	2.89–15.7	< 0.001	3.29	1.52–7.13	0.003
Previous septic shock	2.29	1.33–3.96	0.003	2.39	1.14–5.01	0.02			
Acute kidney injury				4.77	1.94–11.8	< 0.001			
Heart disease	1.78	0.96–3.33	0.07	3.78	1.09–13.1	0.006			
Renal replacement therapy	2.16	1.11–4.21	0.02						
Glycopeptides^5, 6^							3.31	1.33–8.23	0.01
Nitroimidazoles^5, 6^	2.16	1.05–4.45	0.04				3.12	1.07–9.11	0.04
Aminoglycosides^5, 6^				2.28	1.01–5.13	0.05			

*OR* stands for odds ratio, *CI* for confidence interval

^1^Variables in multivariate models were selected by stepwise regression, using a cutoff *p* value of 0.1. The number of patients in the model may be lower than the total number of patients due to missing co-variables in some individuals

^2^The models are not changed and the association with antibiotics is still significant when the variable “intraabdominal bacterial infection” is forced into the model

^3^SAPS2 was not included in the model since it is composed of variables which are presented separately in the model

^4^Within 2 weeks before candidemia (cases) or matched time period (controls)

^5^Within 4 weeks before candidemia (cases) or matched time period (controls)

^6^The association between these classes of antibiotics and candidemia is still significant when the variable “number of antibiotics” is added in the model (independent variables)

Independent risk factors for candidemia within the ICU population included TPN (OR = 6.75, 95%CI 2.89–15.7, *p* < 0.001), acute kidney injury (OR = 4.77, 95%CI 1.94–11.8, *p* < 0.001), heart disease (OR = 3.78, 95%CI 1.09–13.1, *p* = 0.006), previous septic shock (OR = 2.39, 95%CI 1.14–5.01, *p* = 0.02), and exposure to aminoglycosides (OR = 2.28, 95%CI 1.01–5.13, *p* = 0.05).

Independent risk factors for candidemia within the non-ICU population included CVC (OR = 9.77, 95%CI 3.72–25.7, *p* < 0.001), TPN (OR = 3.29, 95%CI 1.52–7.13, *p* = 0.003), exposure to glycopeptides (OR = 3.31, 95%CI 1.33–8.23, *p* = 0.04), and to nitroimidazoles (OR = 3.12, 95%CI 1.07–9.11, *p* = 0.04).

**Fig. 1:**
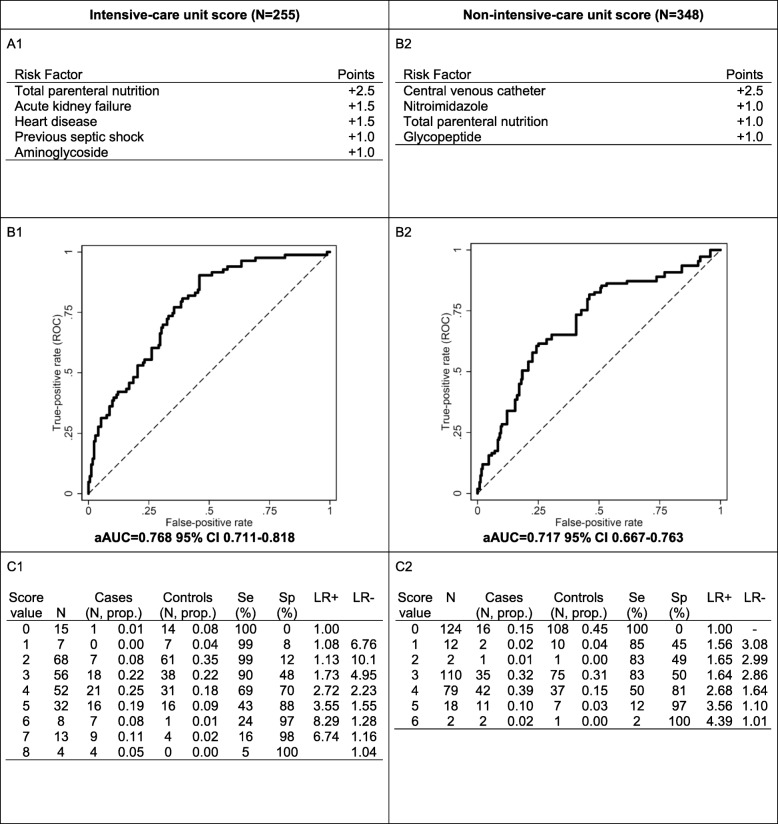
Risk scores for candidemia. Scoring values assigned to each variable (**A1** and **A2**), resulting ROC curves with adjusted areas under the curve (aAUCs, B1 and B2) and single risk performance values (**C1** and **C2**) are shown for patients inside and outside ICU, respectively. Se, Sp, LR+, and LR− stand for sensitivity, specificity, positive and negative likelihood ratios, respectively. The number of patients included in the calculation of score may be lower than the total number of patients due to missing co-variables in some individual patients

Predictive scores for candidemia based on the aforementioned risk factors were developed for both ICU and non-ICU patients (Fig. 1, panel A1 and A2, respectively). The area under the curve (AUC) was 0.768 for ICU patients and 0.717 for non-ICU patients. The optimal cutoff value for the best compromise between sensitivity and specificity was ≥ 4 for ICU patients (sensitivity = 69%, and specificity = 70%) and ≥ 2 for non-ICU patients (sensitivity = 83% and specificity = 49%). Considering a method maximizing efficiency, the optimal cutoff for a better specificity was ≥ 5 for ICU patients (sensitivity = 43%, specificity = 88%) and ≥ 4 for non-ICU patients (sensitivity = 51% and specificity = 81%).

### Risk factors of mortality

Univariate and multivariate analysis of risk factors for death in candidemic patients according to hospital setting are shown in Table 3 and in Table 4, respectively. Independent risk factors for death in the whole population included septic shock (OR = 6.80, 95%CI 2.93–15.8, *p* < 0.001), acute kidney injury (OR = 5.62, 95%CI 2.44–12.9, *p* < 0.001), and the number of antibiotics (OR = 1.43, 95%CI 1.16–1.77 per unit, *p* < 0.001). Age tended to be associated with death (*p* = 0.06). Independent risk factors for death in ICU patients included septic shock (OR = 4.09, 95%CI 1.72–14.0, *p* = 0.003), acute kidney injury (OR = 3.45, 95%CI 1.21–9.90, *p* = 0.02), and the number of antibiotics to which patients were exposed before candidemia (OR = 1.37, 95%CI 1.06–1.75 per unit, *p* = 0.02). Independent risk factors for death in non-ICU patients included acute kidney injury (OR = 11.9, 95%CI 2.47–57.7, *p* = 0.002) and septic shock (OR = 8.70, 95%CI 2.26–33.5, *p* = 0.002).

**Table 3:** Risk factors for death in candidemic patients, according ICU vs non-ICU setting

Characteristics	Whole population			Intensive care			Non-intensive care		
	Death (*n* = 46)	Survival (*n* = 146)	*p*	Death (*n* = 32)	Survival (*n* = 51)	*p*	Death (*n* = 14)	Survival (*n* = 95)	*p*
Age	70 (55–74)	62 (53–73)	0.10	66 (53–73)	59 (52–70)	0.14	73 (68–76)	64 (53–74)	0.12
Underlying medical conditions									
Heart disease	42 (91%)	117 (80%)	0.09	30 (94%)	46 (90%)	0.57	12 (86%)	71 (75%)	0.40
Respiratory disease	11 (24%)	20 (14%)	0.10	9 (28%)	11 (22%)	0.50	2 (14%)	9 (9%)	0.60
Renal failure	32 (70%)	42 (29%)	**< 0.001**	25 (78%)	26 (51%)	**0.02**	7 (50%)	16 (17%)	**0.008**
Liver disease	7 (15%)	12 (9%)	0.20	6 (19%)	6 (12%)	0.38	1 (7%)	7 (7%)	1.00
Central nervous system disease	11 (24%)	19 (13%)	0.08	8 (25%)	6 (12%)	0.12	3 (21%)	13 (14%)	0.40
Diabetes	15 (34%)	34 (23%)	0.20	11 (34%)	12 (24%)	0.28	4 (29%)	22 (23%)	0.70
Solid organ transplant	4 (9%)	5 (3%)	0.15	4 (13%)	2 (4%)	0.16	0 (0%)	3 (3%)	–
Solid cancer	9 (20%)	32 (22%)	0.70	5 (16%)	12 (24%)	0.39	5 (36%)	23 (24%)	0.40
Onco-hematological disease	1 (2%)	9 (6%)	0.30	0 (0%)	1 (2%)	–	1 (7%)	8 (8%)	0.90
Neutropenia	2 (4%)	9 (6%)	0.60	0 (0%)	0 (0%)	–	2 (14%)	(9%)	0.60
Inflammatory disease	6 (13%)	15 (10%)	0.60	3 (9%)	4 (8%)	0.81	3 (21%)	11 (12%)	0.30
Immunosuppression	4 (9%)	6 (4%)	0.20	3 (9%)	2 (4%)	0.32	1 (7%)	4 (4%)	0.60
Pancreatitis	2 (4%)	9 (6%)	0.60	0 (0%)	3 (6%)	–	2 (14%)	6 (6%)	0.30
Bacterial co-infection	41 (89%)	99(68%)	**0.007**	30 (94%)	40 (78%)	0.08	11 (79%)	59 (62%)	0.20
Septic shock concomitant to candidemia	27 (59%)	28 (19%)	**< 0.001**	18 (56%)	13 (25%)	**0.008**	9 (64%)	15 (16%)	**< 0.001**
SAPS2	NA	NA	NA	62 (43–75)	48 (40–66)	0.14	NA	NA	NA
Hospital management and clinical risk factors									
Intensive care Unit	35 (76%)	62 (42%)	**< 0.001**	NA	NA	NA	NA	NA	NA
Extra renal epuration	24 (52%)	20 (14%)	**< 0.001**	20 (63%)	16 (31%)	**0.006**	4 (29%)	4 (4%)	**0.005**
Invasive mechanical ventilation	31 (67%)	44 (30%)	**< 0.001**	29 (91%)	40 (78%)	0.16	2 (14%)	4 (4%)	0.15
Central venous catheter	42 (91%)	128 (88%)	0.60	31 (97%)	49 (96%)	0.85	11 (79%)	79 (84%)	0.60
CVC ablation	33 (72%)	108 (74%)	0.80	25 (78%)	44 (86%)	0.34	8 (57%)	64 (67%)	0.50
Delay between the first day of candidemia and CVC ablation	2 (0–5)	2 (1–4)	0.60	2 (0–3)	2 (1–4)	0.34	5 (2–6)	2 (1–3)	1.00
Total parenteral nutrition	21 (46%)	56 (39%)	0.40	15 (47%)	23 (45%)	0.87	6 (43%)	33 (35%)	0.60
Antiacids	38 (83%)	118 (81%)	0.80	27 (84%)	41 (80%)	0.65	11 (79%)	77 (81%)	0.80
Urinary catheter	40 (87%)	97 (67%)	**0.01**	30 (94%)	47 (92%)	1.00	11 (71%)	50 (53%)	0.20
Surgery before candidemia	18 (39%)	71 (49%)	0.30	14 (44%)	31 (61%)	0.22	4 (29%)	40 (42%)	0.30
Antifungal prophylaxis	6 (13%)	14 (10%)	0.50	5 (16%)	3 (6%)	0.16	1 (7%)	11 (12%)	0.60
Delay of introduction of antifungal therapy	1 (0–2)	2 (0–2)	0.50	1 (−1–2)	2 (0–3)	**0.04**	2 (1–2)	2 (0–2)	0.60
Antibiotics	44 (96%)	130 (89%)	0.20	31 (97%)	48 (94%)	0.57	13 (93%)	82 (86%)	0.50
Number of antibiotics	4 (2–5)	2 (1–3)	**< 0.001**	**4 (3–5)**	**3 (2–4)**	**0.04**	2 (1–3)	2 (1–3)	0.14
Laboratory data									
Leucocytes (.10^3^ /mm^3^)	18 (10–29)	13 (8–20)	**0.009**	21 (12–31)	19 (13–26)	0.61	12 (7–28)	10 (7–17)	0.11
CRP (mg/L)	208 (108–305)	152 (87–246)	**0.04**	167 (80–306)	186 (113–244)	0.59	212 (145–282)	141 (69–247)	**0.03**
PCT (μg/L)	9 (2–40)	3 (1–9)	0.20	8 (2–19)	3 (1–11)	0.18	48 (43–52)	2 (0–6)	0.30
Β-D-glucan (pg/mL)	249 (126–1056)	85 (20–277)	0.40	251 (140–1065)	52 (14–236)	0.47	190 (69–2127)	111 47–451)	0.60
*Candida* species in blood cultures									
*C. albicans*	30 (65%)	84 (58%)		20 (63%)	33 (65%)		10 (71%)	51 (54%)	
*C. glabrata*	5 (11%)	26 (18%)	0.20	3 (9%)	9 (18%)	0.41	2 (14%)	17 (18%)	0.60
*C. parapsilosis*	1 (2%)	18 (12%)	0.08	1 (3%)	3 (6%)	0.62	0	15 (16%)	–
*C. tropicalis*	1 (2%)	5 (3%)	0.60	1 (3%)	3 (6%)	0.62	0	2 (2%)	–
*C. krusei*	3 (7%)	3 (2%)	0.20	3 (9%)	0 (0%)	–	0	3 (3%)	–

*NA* not applicable

**Table 4:** Independent risk factors of death associated with all-cause death in candidemic patients according to the ICU vs non-ICU hospital setting

Risk factors	Whole population^1^ (*N* = 191)			Intensive care unit^1, 2^ (*N* = 83)			Non-ICU^1^ (*N* = 108)		
	OR	95%CI	*p*	OR	95%CI	*p*	OR	95%CI	*p*
Age^2^	1.03	1.00–1.06	0.06						
Acute kidney injury	5.62	2.44–12.9	< 0.001	3.45	1.21–9.90	0.02	11.9	2.47–57.7	0.002
Septic shock concomitant to candidemia	6.80	2.93–15.8	< 0.001	4.09	1.72–14.0	0.003	8.70	2.26–33.5	0.002
Number of antibiotics^3^	1.43	1.16–1.77	< 0.001	1.37	1.06–1.77	0.01			

^1^Variables in multivariate models were selected by stepwise regression, using a cutoff *p* value of 0.1

^2^SAPS2 was not included in the model since it is composed of variables which are presented separately in the model

^3^Per unit (i.e., 1 year for age and one compound for antibiotics, respectively)

## Discussion

This prospective, multicenter, matched case-control study was designed to analyze risk factors for candidemia in both ICU and non-ICU patients. The study included the largest number of candidemic patients reported from a case-control study in the ICU [25] and the second largest sample size for a case-control study outside the ICU [21]. Different risk factors for candidemia were identified in both settings, allowing for targeted risk factor selection.

Because invasive candidiasis is a rare clinical event, previous studies have included cases irrespective of the presence or absence of candidemia [22–24, 27]. Non-candidemic patients can represent up to 30% of cases in some studies [22, 24]. The term “invasive candidiasis” is applied to very differently defined clinical conditions. Some of these, such as post-surgical intra-abdominal candidiasis, require a complex diagnostic approach with clinical and microbiological expertise [35], while others, such as candidemia, represent a clear-cut phenotype. In order to maximize case homogeneity and minimize the risk for misclassification, we considered only patients with candidemia in the present study. Furthermore, we used a matched case-control design, with matching criteria similar to those used in the seminal paper by Wey et al. [25], adding a more stringent matching for the type of surgery. A novelty of the present study is the application of a matched case-control design in ICU patients. The matching criteria aimed at separating risk factors that are specific for candidemia from those that result from prolonged hospitalization [25].

Overall, the study confirms the well-established risk factors for candidemia, such as total parenteral nutrition (the most robust one, which was identified in all studies [10, 21–25, 27]), central venous catheter [10, 23–25, 28], septic shock [21, 22], kidney failure, or renal replacement [10, 23, 25], as well previous exposure to antibiotics (without class specification) [21, 23–25]. The study also highlights the specific risk factors for candidemia that emerge for the ICU and the non-ICU settings, as illustrated by specific patterns of antibiotic exposure, as well as clinical features or medical equipment. For instance, CVC was an independent risk factor for candidemia outside the ICU, probably reflecting its very frequent use (> 90% of patients) in ICU, making it non-discriminant for the determination of the risk of candidemia this setting [22]. In contrast, septic shock was associated with candidemia solely inside the ICU, in accordance with the two studies by Léon et al. [22, 27], reflecting the fact that most patients with such complication are managed in this setting. The other clinical features associated with candidemia solely among ICU patients included heart failure and kidney injury which not previously reported in this setting.

One of the most striking findings of this study was the different patterns of antibiotic exposure associated with candidemia in ICU and non-ICU patients. Glycopeptides and nitroimidazoles were associated with candidemia only outside the ICU. The frequent use of these drugs in the ICU may explain the lack of association in this specific setting. This finding is consistent with a recent study in patients on internal medicine wards, in which glycopeptides were found to be an independent risk factor for candidemia [21]. As intraabdominal bacterial infections were associated with candidemia in the ICU population in univariate analysis (but not in multivariate one), we have forced this variable in the multivariate models for the whole population and the non-ICU one to check for bias. The association between candidemia and glycopeptides/nitroimidazoles remains significant so that these classes of antibiotics can be considered as independent from intraabdominal bacterial infections. In contrast, aminoglycosides were an independent risk factor for candidemia solely in the ICU. These drugs may represent a supplementary risk factor for developing candidemia among ICU patients, who are exposed to multiple other classes of antibiotics (including drugs active against Gram-negative anaerobic bacteria) and/or to glycopeptide antibiotics. Because control matching was performed on a center basis, the associations with antibiotics are not likely to reflect any differences in center’s antibiotic stewardship or empirical treatment strategies.

*Candida* colonization was previously reported as a risk factor for candidemia in some studies [22, 25, 27], but not in others [21, 23, 24]. Colonization was not systematically tested in all patients, thereby limiting the statistical power to detect an association with candidemia. The different practices to monitor *Candida* colonization among centers due to logistic and financial issues may limit its universal use to assess the risk of candidemia. On the other hand, *Candida* colonization, if systematically monitored over time during prolonged hospitalization, may become too frequent to be a discriminant predictor [36]. Corticosteroids and other immunosuppressive drugs were not associated with candidemia in the present study, neither in ICU nor in non-ICU patients. Corticosteroids were inconstantly associated with candidemia in previous studies, possibly due to the lack of standard definitions for high-risk corticosteroid dose and duration of exposure [21–24].

Both the non-ICU and ICU predictive scores for candidemia in this study can be used with relative low cutoff values. The high negative-predictive values associated with low cutoffs can be useful to identify patients in whom the occurrence of candidemia is unlikely, thereby avoiding the use of unnecessary antifungal prophylaxis or empirical/pre-emptive therapy [22, 27]. Alternatively, high positive-predictive values associated with higher cutoffs are applied in other studies for selecting patients who might benefit from empirical/pre-emptive antifungal therapy [21, 23]. In our study, the accuracy and the compromise between sensitivity and specificity is better for the ICU score than for the non-ICU score. The score in the ICU setting could be used both to exclude candidemia (low cutoff) or to detect candidemic patients (high cutoff). The scores should be validated and evaluated in a validation cohort.

This study extends the list of risk factors for candidemia that exert a strong influence on the intestinal microbiota. The gut is the most frequent portal of entry for invasive infection due to *Candida* spp. [37], as a key locus for host-pathogen interactions [38] and a major determinant for the transition from colonization to infection [39]. In mice models, TPN and subsequent enteral deprivation lead to important modifications in the gut microbiota (with a shift of the predominance of Gram-positive Firmicutes to Gram-negative Proteobacteria), alteration in the barrier function of epithelial cells [40], and intestinal inflammation [40, 41]. In mice, antibiotic administration is increasingly shown to exert important and long-lasting alterations on the gut microbiota, which can induce proliferation of pathogenic microorganisms [42]. Administration of drugs such as carbapenems [43], fluoroquinolones [44], and glycopeptides [45], this last one being recognized as independent risk factors for candidemia in the present study, has been associated with increased *Candida* gut colonization in mice, as a probable result of altered relative proportions of anaerobic and aerobic bacteria in the microbiome.

The results from this study are strengthened by a large sample size, with the largest collection of candidemia cases from ICU in a case-control study today and a prospective case-control study design. Yet, control matching implies the use of conditional regression models, which limits statistical power. Furthermore, the number of controls per case is smaller than in a cohort study, thereby limiting predictive score performance. The ICU setting and surgery were used as matching criteria and thus were not assessable as risk factor in this study. While our study suggests that risk assessment and scoring need to account for the hospital setting (ICU versus non-ICU), larger studies allowing for scores in even more specific groups of patients (such as medical, surgical, onco-hematological patients) would further improve risk prediction.

## Conclusion

We show that risk factors for candidemia are different among patients hospitalized inside and outside ICUs. Specific patterns of antibiotic exposure are emerging as novel risk factors for candidemia. These include aminoglycosides for patients hospitalized within the ICU and glycopeptides and nitroimidazoles for patients hospitalized outside the ICU. Weighted scores predictive of candidemia can be built based on these risks. An improved prediction of the risk of candidemia may contribute to guide targeted preventive and therapeutic antifungal strategies.

## Data Availability

All data generated or analyzed in this study are included in this published article, and the datasets are available from the corresponding author within the limits imposed by ethical and legal dispositions.

## References

[1] Wisplinghoff H, Bischoff T, Tallent SM, Seifert H, Wenzel RP, Edmond MB (2004). Nosocomial bloodstream infections in US hospitals: analysis of 24,179 cases from a prospective nationwide surveillance study. Clin Infect Dis.

[2] Arendrup MC, Bruun B, Christensen JJ, Fuursted K, Johansen HK, Kjaeldgaard P, Knudsen JD, Kristensen L, Moller J, Nielsen L (2011). National surveillance of fungemia in Denmark (2004 to 2009). J Clin Microbiol.

[3] Cleveland AA, Farley MM, Harrison LH, Stein B, Hollick R, Lockhart SR, Magill SS, Derado G, Park BJ, Chiller TM (2012). Changes in incidence and antifungal drug resistance in candidemia: results from population-based laboratory surveillance in Atlanta and Baltimore, 2008-2011. Clin Infect Dis.

[4] Lortholary O, Renaudat C, Sitbon K, Madec Y, Denoeud-Ndam L, Wolff M, Fontanet A, Bretagne S, Dromer F, French Mycosis Study G (2014). Worrisome trends in incidence and mortality of candidemia in intensive care units (Paris area, 2002-2010). Intensive Care Med.

[5] Lamoth F, Lockhart SR, Berkow EL, Calandra T (2018). Changes in the epidemiological landscape of invasive candidiasis. J Antimicrob Chemother.

[6] Chapman B, Slavin M, Marriott D, Halliday C, Kidd S, Arthur I, Bak N, Heath CH, Kennedy K, Morrissey CO (2017). Changing epidemiology of candidaemia in Australia. J Antimicrob Chemother.

[7] Paiva JA, Pereira JM, Tabah A, Mikstacki A, de Carvalho FB, Koulenti D, Ruckly S, Cakar N, Misset B, Dimopoulos G (2016). Characteristics and risk factors for 28-day mortality of hospital acquired fungemias in ICUs: data from the EUROBACT study. Crit Care.

[8] Kett DH, Azoulay E, Echeverria PM, Vincent JL, Extended Prevalence of infection in ICUSGoI (2011). Candida bloodstream infections in intensive care units: analysis of the extended prevalence of infection in intensive care unit study. Crit Care Med.

[9] Colombo AL, Guimaraes T, Sukienik T, Pasqualotto AC, Andreotti R, Queiroz-Telles F, Nouer SA, Nucci M (2014). Prognostic factors and historical trends in the epidemiology of candidemia in critically ill patients: an analysis of five multicenter studies sequentially conducted over a 9-year period. Intensive Care Med.

[10] Blumberg Henry M., Jarvis William R., Soucie J. Michael, Edwards Jack E., Patterson Jan E., Pfaller Michael A., Rangel‐Frausto M. Sigfrido, Rinaldi Michael G., Saiman Lisa, Wiblin R. Todd, Wenzel Richard P. (2001). Risk Factors for Candidal Bloodstream Infections in Surgical Intensive Care Unit Patients: The NEMIS Prospective Multicenter Study. Clinical Infectious Diseases.

[11] Vincent JL, Rello J, Marshall J, Silva E, Anzueto A, Martin CD, Moreno R, Lipman J, Gomersall C, Sakr Y (2009). International study of the prevalence and outcomes of infection in intensive care units. JAMA.

[12] Blot SI, Vandewoude KH, Hoste EA, Colardyn FA (2002). Effects of nosocomial candidemia on outcomes of critically ill patients. Am J Med.

[13] Gudlaugsson O, Gillespie S, Lee K, Vande Berg J, Hu J, Messer S, Herwaldt L, Pfaller M, Diekema D (2003). Attributable mortality of nosocomial candidemia, revisited. Clin Infect Dis.

[14] Leleu G, Aegerter P, Guidet B, College des Utilisateurs de Base de Donnees en R (2002). Systemic candidiasis in intensive care units: a multicenter, matched-cohort study. J Crit Care.

[15] Kim PW, Perl TM, Keelaghan EF, Langenberg P, Perencevich EN, Harris AD, Song X, Roghmann MC (2005). Risk of mortality with a bloodstream infection is higher in the less severely ill at admission. Am J Respir Crit Care Med.

[16] Kollef M, Micek S, Hampton N, Doherty JA, Kumar A (2012). Septic shock attributed to Candida infection: importance of empiric therapy and source control. Clin Infect Dis.

[17] Playford EG, Eggimann P, Calandra T (2008). Antifungals in the ICU. Curr Opin Infect Dis.

[18] Lockhart SR, Iqbal N, Cleveland AA, Farley MM, Harrison LH, Bolden CB, Baughman W, Stein B, Hollick R, Park BJ (2012). Species identification and antifungal susceptibility testing of Candida bloodstream isolates from population-based surveillance studies in two U.S. cities from 2008 to 2011. J Clin Microbiol.

[19] Shields RK, Nguyen MH, Press EG, Cumbie R, Driscoll E, Pasculle AW, Clancy CJ (2015). Rate of FKS mutations among consecutive Candida isolates causing bloodstream infection. Antimicrob Agents Chemother.

[20] Shields RK, Nguyen MH, Clancy CJ (2015). Clinical perspectives on echinocandin resistance among Candida species. Curr Opin Infect Dis.

[21] Falcone M, Tiseo G, Tascini C, Russo A, Sozio E, Raponi G, Rosin C, Pignatelli P, Carfagna P, Farcomeni A (2017). Assessment of risk factors for candidemia in non-neutropenic patients hospitalized in internal medicine wards: a multicenter study. Eur J Intern Med.

[22] Leon C, Ruiz-Santana S, Saavedra P, Almirante B, Nolla-Salas J, Alvarez-Lerma F, Garnacho-Montero J, Leon MA, Group ES (2006). A bedside scoring system (“Candida score”) for early antifungal treatment in nonneutropenic critically ill patients with Candida colonization. Crit Care Med.

[23] Ostrosky-Zeichner L, Sable C, Sobel J, Alexander BD, Donowitz G, Kan V, Kauffman CA, Kett D, Larsen RA, Morrison V (2007). Multicenter retrospective development and validation of a clinical prediction rule for nosocomial invasive candidiasis in the intensive care setting. Eur J Clin Microbiol Infect Dis.

[24] Playford EG, Lipman J, Jones M, Lau AF, Kabir M, Chen SC, Marriott DJ, Seppelt I, Gottlieb T, Cheung W (2016). Problematic dichotomization of risk for intensive care unit (ICU)-acquired invasive candidiasis: results using a risk-predictive model to categorize 3 levels of risk from a multicenter prospective cohort of Australian ICU patients. Clin Infect Dis.

[25] Wey SB, Mori M, Pfaller MA, Woolson RF, Wenzel RP (1989). Risk factors for hospital-acquired candidemia. A matched case-control study. Arch Intern Med.

[26] Bassetti M, Mikulska M, Viscoli C (2010). Bench-to-bedside review: therapeutic management of invasive candidiasis in the intensive care unit. Crit Care.

[27] Leon C, Ruiz-Santana S, Saavedra P, Galvan B, Blanco A, Castro C, Balasini C, Utande-Vazquez A, Gonzalez de Molina FJ, Blasco-Navalproto MA (2009). Usefulness of the “Candida score” for discriminating between Candida colonization and invasive candidiasis in non-neutropenic critically ill patients: a prospective multicenter study. Crit Care Med.

[28] Shahin J, Allen EJ, Patel K, Muskett H, Harvey SE, Edgeworth J, Kibbler CC, Barnes RA, Biswas S, Soni N (2016). Predicting invasive fungal disease due to Candida species in non-neutropenic, critically ill, adult patients in United Kingdom critical care units. BMC Infect Dis.

[29] Lacroix C, Gicquel A, Sendid B, Meyer J, Accoceberry I, Francois N, Morio F, Desoubeaux G, Chandenier J, Kauffmann-Lacroix C (2014). Evaluation of two matrix-assisted laser desorption ionization-time of flight mass spectrometry (MALDI-TOF MS) systems for the identification of Candida species. Clin Microbiol Infect.

[30] Sendid B, Ducoroy P, Francois N, Lucchi G, Spinali S, Vagner O, Damiens S, Bonnin A, Poulain D, Dalle F (2013). Evaluation of MALDI-TOF mass spectrometry for the identification of medically-important yeasts in the clinical laboratories of Dijon and Lille hospitals. Med Mycol.

[31] Pittet D, Monod M, Suter PM, Frenk E, Auckenthaler R (1994). Candida colonization and subsequent infections in critically ill surgical patients. Ann Surg.

[32] Pepe MS, Fan J, Seymour CW (2013). Estimating the receiver operating characteristic curve in studies that match controls to cases on covariates. Acad Radiol.

[33] Youden WJ (1950). Index for rating diagnostic tests. Cancer.

[34] Zweig MH, Campbell G (1993). Receiver-operating characteristic (ROC) plots: a fundamental evaluation tool in clinical medicine. Clin Chem.

[35] Monitoring of 1,3-Beta-D-Glucan in High-Risk Surgical ICU Patients for Early Diagnosis of Invasive Candidiasis: a Prospective Study of the Fungal Infection Network of Switzerland (FUNGINOS). Poster Presentation # M-1071. 50th ICAAC, September 12–15 2010.

[36] Charles PE, Dalle F, Aube H, Doise JM, Quenot JP, Aho LS, Chavanet P, Blettery B (2005). Candida spp. colonization significance in critically ill medical patients: a prospective study. Intensive Care Med.

[37] Reagan DR, Pfaller MA, Hollis RJ, Wenzel RP (1990). Characterization of the sequence of colonization and nosocomial candidemia using DNA fingerprinting and a DNA probe. J Clin Microbiol.

[38] Netea MG, Joosten LA, van der Meer JW, Kullberg BJ, van de Veerdonk FL (2015). Immune defence against Candida fungal infections. Nat Rev Immunol.

[39] Lagunes L, Rello J (2016). Invasive candidiasis: from mycobiome to infection, therapy, and prevention. Eur J Clin Microbiol Infect Dis.

[40] Demehri FR, Barrett M, Ralls MW, Miyasaka EA, Feng Y, Teitelbaum DH (2013). Intestinal epithelial cell apoptosis and loss of barrier function in the setting of altered microbiota with enteral nutrient deprivation. Front Cell Infect Microbiol.

[41] Harris JK, El Kasmi KC, Anderson AL, Devereaux MW, Fillon SA, Robertson CE, Wagner BD, Stevens MJ, Pace NR, Sokol RJ (2014). Specific microbiome changes in a mouse model of parenteral nutrition associated liver injury and intestinal inflammation. PLoS One.

[42] Antonopoulos DA, Huse SM, Morrison HG, Schmidt TM, Sogin ML, Young VB (2009). Reproducible community dynamics of the gastrointestinal microbiota following antibiotic perturbation. Infect Immun.

[43] Samonis G, Maraki S, Leventakos K, Spanaki AM, Kateifidis A, Galanakis E, Tselentis Y, Falagas ME, Mantadakis E (2006). Comparative effects of ertapenem, imipenem, and meropenem on the colonization of the gastrointestinal tract of mice by Candida albicans. Med Mycol.

[44] Samonis G, Kofteridis DP, Maraki S, Alegakis D, Mantadakis E, Papadakis JA, Gikas AH, Falagas ME (2005). Levofloxacin and moxifloxacin increase human gut colonization by Candida species. Antimicrob Agents Chemother.

[45] Samonis G, Maraki S, Barbounakis E, Leventakos K, Lamaris G, Rovithi M, Hatjiioannou I, Potolidis E, Tselentis Y, Mantadakis E (2006). Effects of vancomycin, teicoplanin, linezolid, quinupristin-dalfopristin, and telithromycin on murine gut colonization by Candida albicans. Med Mycol.

